# Immune-Related Gene Expression Profiling of Broiler Chickens Fed Diets Supplemented with Vinification Byproducts: A Valorization Approach II

**DOI:** 10.3390/ani11113038

**Published:** 2021-10-22

**Authors:** Alexandros Mavrommatis, Panagiotis E. Simitzis, Panagiota Kyriakaki, Elisavet Giamouri, Eleni D. Myrtsi, Epameinondas Evergetis, Katiana Filippi, Harris Papapostolou, Sofia D. Koulocheri, Athanasios C. Pappas, Apostolis Koutinas, Serkos A. Haroutounian, Eleni Tsiplakou

**Affiliations:** 1 Laboratory of Nutritional Physiology and Feeding, Department of Animal Science, School of Animal Biosciences, Agricultural University of Athens, Iera Odos 75, 11855 Athens, Greece; mavrommatis@aua.gr (A.M.); kyriakaki@aua.gr (P.K.); egiamouri@aua.gr (E.G.); elenamirtsi@aua.gr (E.D.M.); epaev@aua.gr (E.E.); skoul@aua.gr (S.D.K.); apappas@aua.gr (A.C.P.); sehar@aua.gr (S.A.H.); 2 Laboratory of Animal Breeding & Husbandry, Department of Animal Science, Agricultural University of Athens, Iera Odos 75, 11855 Athens, Greece; pansimitzis@aua.gr; 3 Laboratory of Food Process Engineering, Department of Food Science and Human Nutrition, Agricultural University of Athens, Iera Odos 75, 11855 Athens, Greece; filippi@aua.gr (K.F.); harris_papapostolou@yahoo.gr (H.P.); akoutinas@aua.gr (A.K.)

**Keywords:** bursa of Fabricius, flavonoids, grape pomace, grape stems, liver, polyphenols, spleen, wine lees, wine yeast cells

## Abstract

**Simple Summary:**

The valorization of grape byproducts appears to be a sustainable bioeconomic strategy that could promote the substitution of synthetic with natural antioxidant compounds in the food and feed industry. The nutritional physiology of broiler chickens requires special attention to the interactions between nutrients and antioxidant mechanisms since the stressor signals of factory farming could impair the immune status, resulting in detrimental effects on broilers’ performance. The objective of this study was to assess the inclusion of grape byproducts (grape pomace, wine lees, and stem extract) on the transcriptional profiling of genes regulating the immune system in the liver, bursa of Fabricius, and spleen. The dietary supplementation of grape byproducts rich in polyphenolic compounds decreased the mRNA levels of the predominant pro-inflammatory receptor in the liver, while in the spleen, the stem extract diet upregulated the aforementioned receptor. Upregulation of interleukin 8 was observed in the bursa of Fabricius and spleen of the stems extract-fed broilers. Although grape byproducts depicting a sustainable source of bioactive compounds with vast antioxidant potential, there were unveiled preliminary insights for immune stimulation at the transcriptional level.

**Abstract:**

The valorization of vinification byproducts portrays a promising bioprocess for the enrichment of animals’ diet with bioactive compounds, such as polyphenols, which could regulate the immune response. Therefore, the impact of dietary grounded grape pomace (GGP), wine lees extract (WYC), and grape stem extract (PE) on the relative transcript level of immune related genes of broiler chickens were examined. Two hundred forty, one-day-old as hatched (male/female) chicks (Ross 308) were allocated to four dietary groups, with four replicate pens each with 15 birds. Birds were fed either a basal diet (CON) or the basal diet supplemented with 2.5% GGP, or 0.2% WYC, or 0.1% PE for 42 d. The relative expression of immune-related genes was investigated using a real-time PCR platform. The mRNA levels of Toll-like Receptor 4 (*TLR4)* were downregulated (*p* = 0.039) in the liver of broilers fed the GGP-containing diet compared to the CON, while in the spleen of PE-fed broilers, *TLR4* was significantly upregulated (*p* = 0.043). The mRNA levels of interleukin 8 (*IL8)* tended to upregulate (*p* = 0.099) in the bursa of Fabricius and were significantly increased (*p* = 0.036) in the spleen of broilers fed the PE diet. Vinification byproducts depict a promising sustainable source of polyphenols for the poultry feed industry, but more research is needed under field conditions.

## 1. Introduction

*Vitis vinifera* (common grape vine) appears to be the world’s largest fruit crop, with 71% being used for winemaking [1]. Approximately 62% of global wine production is located within the Mediterranean basin area [1]. The leftovers of the wine process are composed of stems, the woody part of grapevine, and grape pomace; the solid residue of vinification consists of skins, stems, and seeds [2]. These wastes are usually discarded into nearby open fields for biodegradation, polluting the environment and the water reservoirs [3], while plenty of desirable bioactive compounds are wasted as well [4]. Numerous studies have highlighted the potential of these byproducts for use as plant-originated feed additives that are primarily rich in a broad diversity of polyphenols [5,6].

In our preliminary study, we investigated the polyphenolic profile of three vinification byproducts (grape pomace, stems extract, and wine lees) and their potential as feed additives in broiler chickens [7]. Interestingly, the prevailing compounds of grape byproducts, such as gallic acid, resveratrol, catechin, caftaric acid, quercetin, and procyanidins, slightly enhanced the blood antioxidants mechanisms and improved the breast muscle oxidative status [7]. However, the inclusion of stem extract unveiled some warning flags which require further elucidation. More specifically, the mRNA abundances of NADPH oxidase 1 (*NOX1*) and *NOX3* were tended to increase in the liver of broilers fed with the stem extract, generating concerns regarding possible adverse effects on organism homeostasis [7]. The NADPH oxidases are considered to be the only enzymes whose principal role is to generate superoxide and consequently, other reactive oxygen species (ROS) [8] that could firmly trigger the immune regulation through nuclear factor kappa B (*NF-kB*) [9,10,11]. Several recent lines of evidence reveal that the stimulation of *NF-kB* through the Toll-like receptor 4 (*TLR4*) can be attributed to ROS, such as superoxide and hydrogen peroxide [9]. The activation of the *TLR4-NFkB* pathway leads to increased transcription of genes related to innate immunity and pro-inflammatory responses that can further burden broilers’ performance under factory farming conditions [12]. The activation of the immune response constitutes a significant dietary energy loss since immune regulation requires vast amounts of energy and nutrients [13]. Thus, the inclusion of novel feed additives and feedstuffs should be holistically investigated, aiming to broadly assess their impact.

Although the immunomodulatory effect of polyphenols in the human diet and treated cell lines have been extensively studied, supporting the theory that these bioactive compounds could inhibit inflammation by downregulating the pro-inflammatory cytokines [14,15], scarce information exists about their influence on productive animals. It is worth mentioning that the interaction of the absorbed polyphenols could either protectively or harmfully interact with the intestinal immune system depending on their polymerization and oxidation rate [16]. Still, the effect of polyphenols in immune system regulation has been majorly studied using pure, individual substances in which the synergetic or antagonistic potential of naturally derived ingredients is overlooked.

Considering that issue, this study aimed to evaluate the impact of three winery byproducts (grape pomace, stem extract, and wine lees) on the transcriptional profiling of immune-related genes in the liver, spleen, and bursa of Fabricius of broiler chickens.

## 2. Materials and Methods

### 2.1. Broilers’ Trial

The experimental procedure of the present study continued the analytical approach initiated in our preliminary work [7]. Animals, diets, and experimental design have been previously presented [7]. In brief, two hundred forty (*n* = 240) as hatched (male/female), 1-day-old, Ross 308 broilers were assigned to 4 experimental treatments for 42 days. Each treatment had four floor replicate cages of 15 broilers each [body weight 44.23 ± 0.90 (S.D.) g]. Each replicate was allocated to a clean floor cage (2 m^2^), and the birds were raised on wheat straw litter. The house environmental conditions were controlled according to commercial recommendations, and the heat was provided with a heating infrared lamp per pen.

### 2.2. Vinification Byproducts Processing and Diets Formulation

The processing of the vinification by-products has been previously described by Mavrommatis et al. [7]. In the control (CON) group, broilers were fed a basal diet based on corn and soybean meal [7]. In the GGP group, grounded grape pomace was added to the starter, grower, and finisher diet at a level of 2.5% (25 g/kg feed). In the WYC group, dried wine lees (approximately 62.5% yeast cells) were added to the starter, grower, and finisher diet at a level of 0.2% (2 g/kg feed). In the PE group, an extract derived from grape stems using soluble starch for its inclusion (10% pure phenolic extract) was added to the starter, grower, and finisher diet at a level of 0.1% (1 g/kg feed). The diet and chemical composition are also available by Mavrommatis et al. [7].

### 2.3. Determination of Total Antioxidant Capacity of Vinification Byproducts

Total antioxidant capacity was triplicately estimated using DPPH and FRAP methods according to Myrtsi et al. [17]. Briefly, 30 µL of each byproduct extract was incubated with 175 µL of 0.1 M methanolic solution of DPPH for 40 min at 25 °C. Then, the absorbance was measured at 515 nm against a standard curve generated using Trolox at room temperature. The determination of the reducing capacity (FRAP) of byproduct extract was performed by monitoring the reduction of Fe^3+^ into Fe^2+^ using 10 mM of 2,4,6-tripyridyl-s-triazine (TPTZ) at 593 nm into an acetate buffer (0.3 M pH 3.6) at 37 °C.

### 2.4. Determination of Total b-1,3-1,6-Glucans of Wine Lees

Wine lees b-glucans were isolated and determined according to Nitschke et al. [18] using a spectrophotometric assay at 523 nm and Congo red dye (0.08 g dye in 100 mL buffer) into 0.2 M citric acid/sodium hydroxide buffer (pH 7).

### 2.5. Determination of Performance Parameters

Bodyweight (BW) was recorded on the first day of the experimental period and at the end of each feeding phase (starter 10th, grower 24th, and finisher 42nd). Feed intake was recorded, and the feed conversion ratio (FCR) was calculated accordingly.

### 2.6. Sample Collection

On day 42 of age (end of the experiment), 32 broilers (8 per treatment and two per replicate pen) were randomly selected and sacrificed. Liver tissue, spleen, and bursa of Fabricius were carefully excised and immediately snap-frozen and subsequently stored at −80 °C for further analyses.

### 2.7. Molecular Analysis

#### 2.7.1. RNA Isolation and cDNA Synthesis

Total RNA was isolated from the liver tissue, spleen, and bursa of Fabricius samples of broilers separately using TriFast (VWR International, Radnor, PEN, Radnor, PA, USA) according to the manufacturer’s instructions. Six micrograms of individually extracted RNA was cured with 2 U of Turbo DNAse (Invitrogen, CA, USA) for 1 h at 37 °C. After DNase treatment, RNA was washed twice using a mixture of 1× vol Phenol:Chloroform:Isoamyl alcohol (25:24:1 *v*/*v*), and then RNA was precipitated by adding 2.5× vol cold ethanol and 0.1× vol 3M sodium acetate at 13,000 g for 30 min at 4 °C.

Pure RNA was used as a template for a qualitative Taq polymerase PCR protocol using glyceraldehyde 3-phosphate dehydrogenase (GAPDH) to confirm (in agarose gel) the absence of any DNA contamination against a positive control (*Gallus gallus* genomic DNA). The integrity of the obtained RNAs was assessed with an agarose gel (3%) showing minor or no RNA hydrolysis. The quantity of RNA was estimated via ng/μL using a microvolume spectrophotometer (NanoDrop ND-1000), and purity was determined by the ratios A260/A280 and A260/A230. Finally, 500 ng of pure RNA was reversely transcribed as previously described [19].

#### 2.7.2. Primers’ Design

A pair of primers specific for mitogen-activated protein kinase (*MAPK*) gene were designed using Geneious software (Biomatters, Ltd., Auckland, New Zealand) according to the respective *Gallus gallus* gene coding sequences (CDS in GenBank) (Table 1). Additionally, a set of primers specific for *GAPDH*, beta-actin (*ACTB*), Toll-like receptors 4 (*TLR4*), nuclear factor kappa B (*NFKB*), tumor necrosis factor (*TNF*), translation initiation factor IF-1 (*INFA*), interferon gamma (*INFG*), interleukin 1 Beta (*IL1B*), interleukin 2 (*IL2*), interleukin 6 (*IL6*), C-X-C motif chemokine ligand 8 (*IL8*), and interleukin 18 (*IL18*), which have been previously initiated were used (Table 1). The specificity of each pair of primers was tested through the dissociation curves, and the amplification products were subjected to agarose gel (2%) electrophoresis to confirm the production of a single amplicon per reaction.

#### 2.7.3. Real-Time Quantitative PCR

The relative transcript levels of the target genes were assayed using a StepOnePlus^TM^ Real-Time PCR System (Applied Biosystems, Foster City, CA, USA) and SYBR Select Master Mix (Applied Biosystems, Austin, TX, USA) using target-specific primers at a final concentration of 0.2 μM each and one μL of each cDNA as a template as previously described by Tsiplakou et al. [19]. PCR program initiated at 95 °C for 8 min, followed by 40 cycles of 95 °C for 15 s and 60 °C for one min. The geometrical mean of *GAPDH* and *ACTB* were used to normalize the cDNA template concentrations as has been previously indicated [20]. The relative expression levels of the target genes were performed as described by Mavrommatis et al. [21], while PCR efficiency was determined by employing the linear regression method on the log (fluorescence) per cycle number (ΔRn) using the LinRegPCR software applying the default settings [22].

### 2.8. Statistics

The dataset was explored with the SPSS.IBM software, and the results are depicted as means ± standard error of means (SEM). For broilers’ growth performance, each experimental unit consisted of the replicate pen while for the molecular analyses the experimental unit considered the animal. Dietary effects were monitored using one-way ANOVA followed by Tukey’s test. GraphPad Prism 6.0 (2012) software was applied to simplify the visualization of the obtained results using interleaved bars. Statistical significance was set at *p* ≤ 0.05. Gender was not included in the statistical model as has been previously justified [7].

Discriminant plots were generated (independent-together method) regarding the relative transcript levels of immune-related genes to investigate their classification amongst the four dietary groups (CON, GGP, WYC, and PE). Wilk’s lambda (λ) criterion was used for assessing discriminant functions. Ten, eleven, and eleven variables were entered to create three models to distinguish the thirty-two samples of each case in the liver, bursa of Fabricius, and spleen, respectively (4 dietary groups × 4 pen replicates/dietary group × 2 animal/pen).

## 3. Results

### 3.1. Grape Byproducts Total Antioxidant Capacity and Wine Lee b-Glucan Content

The total antioxidant capacity of grape pomace (GGP), dried wine lees extract (WYC), and grape stem extract (PE) is presented in Table 2. The total antioxidant capacity of WYC and PE extracts was higher compared to GGP, considering both FRAP and DPPH methods. In the wine lees (WYC) extract, the total b-1,3-1,6-glucans were determined at 7.3% of dried product, which practically means that the WYC diet was supplemented with 146 mg of b-glucans/kg.

### 3.2. Broilers’ Performance

The effects of dietary vinification byproducts on broilers’ performance have been previously reported by Mavrommatis et al., [7]. Briefly, no significant alterations were observed for growth performance, feed intake, and feed conversion rate. However, the mortality rate tended to increase (*p* = 0.079) in the WYC group compared to CON (5% vs. 0%).

### 3.3. Relative Transcript Levels of Genes Regulating the Immune System in Liver

**Figure 1:**
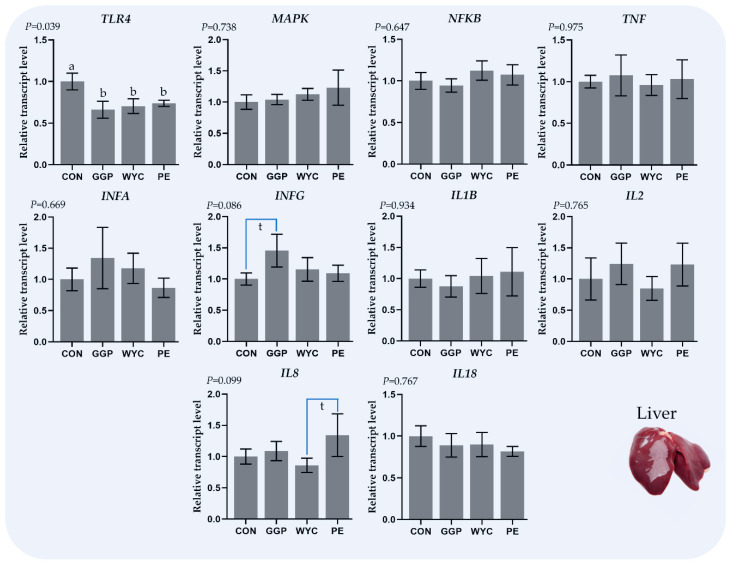
Relative transcript levels (means ± SEM) of immune-related genes in the liver of 42-day-old broilers. Bars with different letter (a, b) amongst the treatments differ significantly (*p* ≤ 0.05) while t represents 0.05 < *p*-value < 0.10.

In Figure 1, the effects of feeding grounded grape pomace (GGP), wine lees (WYC), and grape stem extract (PE) on the relative expression of genes involved in the immune system in the liver of broilers are presented. The relative transcript levels of *TLR4* were significantly downregulated (*p* = 0.039) in the liver of grape byproducts-fed broilers compared to the CON (Figure 1). The relative transcript levels of *INFG* tended to increase (*p* = 0.086) in the GGP-fed broilers compared to the CON (Figure 1). Finally, the relative transcript levels of *IL8* tended to increase (*p* = 0.099) in the PE- compared to the WYC-fed broilers (Figure 1).

### 3.4. Relative Transcript Levels of Genes Regulating the Immune System in the Bursa of Fabricius

**Figure 2:**
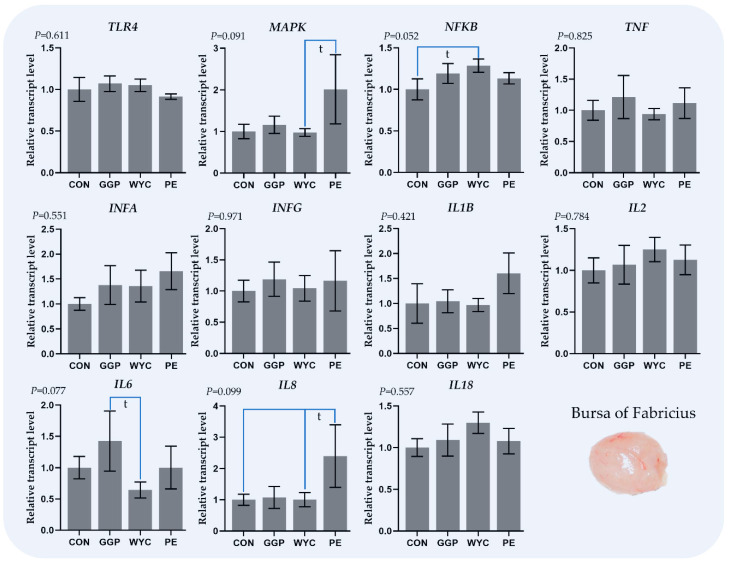
Relative transcript levels (means ± SEM) of immune-related genes in the bursa of Fabricius of 42-day-old broilers. Bars with different letter (a, b) amongst the treatments differ significantly (*p* ≤ 0.05), while t represents 0.05 < *p*-value < 0.10.

In Figure 2, the effects of feeding grounded grape pomace (GGP), wine lees extract (WYC), and grape stem extract (PE) on the relative expression of genes involved in the immune system in the bursa of Fabricius are presented. The relative transcript levels of *MAPK* were tended to increase (*p* = 0.091) in the bursa of Fabricius of PE compared to the WYC dietary supplemented broilers (Figure 2). The relative transcript levels of *NFKB* were tended to increase (*p* = 0.052) in the WYC-fed broilers compared to the controls (Figure 2). On the other hand, the relative transcript levels of *IL6* tended to decrease (*p* = 0.077) in the bursa of Fabricius of WYC compared to the GGP fed broilers (Figure 2). Finally, the relative transcript levels of *IL8* tended to increase (*p* = 0.099) in the PE compared to the WYC and CON groups (Figure 2).

### 3.5. Relative Transcript Levels of Genes Regulating the Immune System in the Spleen

**Figure 3:**
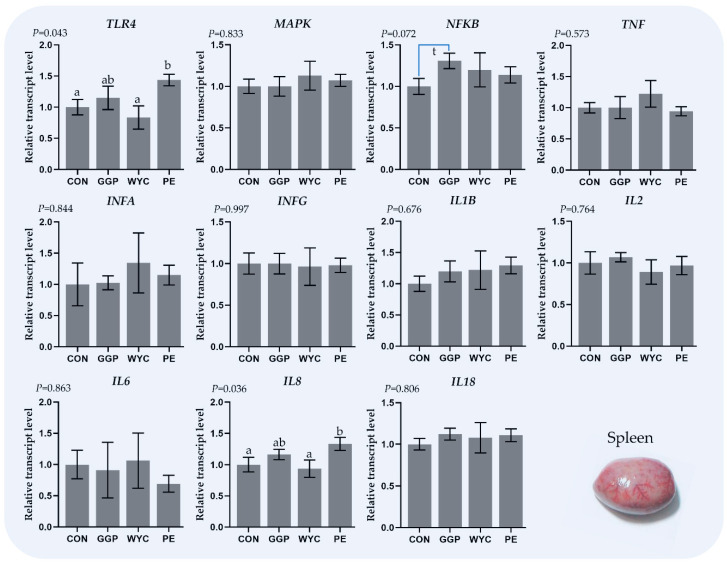
Relative transcript levels (means ± SEM) of immune-related genes in the spleen of 42-day-old broilers. Bars with different letter (a, b) amongst the treatments differ significantly (*p* ≤ 0.05), while t represents 0.05 < *p*-value < 0.10.

In Figure 3, the effects of feeding grounded grape pomace (GGP), wine lees extract (WYC), and grape stem extract (PE) on the relative expression of genes involved in the immune system in the spleen of broilers are presented. The relative transcript levels of *TLR4* were significantly increased (*p* = 0.043) in the spleen of PE compared to that of the WYC and CON group (Figure 3). The relative transcript levels of *NFKB* tended to increase (*p* = 0.072) in the GGP-fed broilers compared to the controls (Figure 3). Finally, the relative transcript levels of *IL8* were significantly increased (*p* = 0.036) in the PE compared to the WYC and CON-fed broilers (Figure 3).

**Figure 4:**
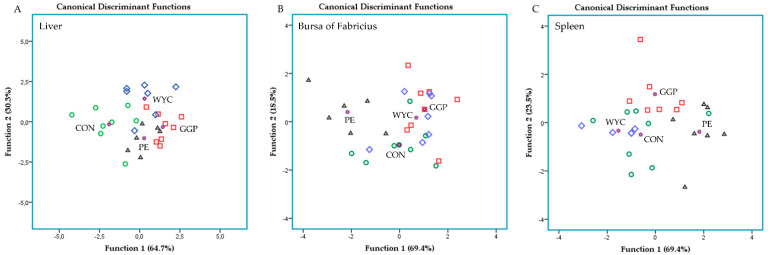
Plots depicting the discrimination of the four dietary groups (CON green, GGP red, WYC blue, and PE black) based on the pooled data of the (**A**) liver, (**B**) bursa of Fabricius, and (**C**) spleen relative transcript levels of immune-related genes.

Discriminant analyses were also applied to the pooled data of liver, bursa of Fabricius, and spleen relative transcript levels to establish those variables capable of distinguishing and classifying samples among the four dietary groups (Figure 4). The proportions of the samples that were correctly classified were 72.4%, 62.1%, and 85.2% for liver, bursa of Fabricius, and spleen, respectively. Wilks’ lambda (λ) was reported at 0.163 for Function 1 (*p* = 0.147) and 0.466 for Function 2 (*p* = 0.591), while the relative transcript levels of *TLR4* was the variable that contributed the most in the liver model due to the downregulation that was observed in the grape-fed groups (Figure 4A). Although the plot supported a moderate discrimination in the case of the liver, the limited dataset underperformed its significance. Wilks’ lambda (λ) was reported at 0.212 for Function 1 (*p* = 0.529) and 0.550 for Function 2 (*p* = 0.907) in the bursa of Fabricius model (Figure 4B). In the spleen model, the Wilks’ lambda (λ) were observed at 0.196 for Function 1 (*p* = 0.609) and 0.519 for Function 2 (*p* = 0.911), while the relative transcript levels of *IL8* was the variable that contributed the most due to the upregulation that was observed in the PE-fed broilers (Figure 4C).

## 4. Discussion

The aim of the present study was to evaluate the transcriptional profiling of immune-related genes of broilers fed diets supplemented with vinification byproducts. Broilers performed well with no major difference between the control group and those fed the vinification byproducts. However, some mRNA levels of pro-inflammatory pathways were upregulated. The numerical increase of mortality was not observed in the PE-fed broilers where the immune stimulation was found triggered based on the transcriptional profiling. Thus, it is plausible to assume that there was not any considerable detrimental effect of the dietary interventions even if some mRNA levels of pro-inflammatory pathways were upregulated. However, it remains an open question, which mechanisms upregulated these transcriptions.

Previous studies have trumpeted the immune-enhancing properties of grape pomace based only on a positive response in the IgM in the primary titer of broilers fed with grape pomace [30,31]. Notably, Hasted et al. [32] summarized eleven studies focused on the effect of grape byproducts on poultry immunity. Interestingly, 9 out of 11 studies did not observe any effect on immune regulation, 2 out of 11 demonstrated a significant improvement in antibody levels, while there were no data about their effect on the pro-inflammatory regulation. Thus, the pronounced immune-enhancing properties of grape derivatives in livestock remain questionable due to a lack of relevant evidence.

In our study, the transcriptional levels of *TLR4* in the liver of broilers fed the three grape byproducts were significantly downregulated. Rahimifard et al. [33] reported the regulation properties of polyphenols with a special focus on resveratrol on *TLR4* cascade. On the other hand, in our study, the expression of *TLR4* in the spleen of broilers fed the PE diet was upregulated. Still, in the same dietary group, a tendency for increased mRNA levels of *NOX1* and *NOX3* was observed [7]. Considering that NADPH oxidases primarily form superoxide anions, it is plausible to assume that higher ROS formation may have triggered TLR4s in the spleen of PE-fed broilers [8,9]. However, the liver is considered to be equipped with a more efficient antioxidative system and be more competent in dietary antioxidant accumulation compared to the spleen, which may neutralize the detrimental ROS before they trigger the *TLR4* pathway [34,35]. Resveratrol has been linked to an improvement of mice’s immune responses by stimulating the synthesis of Th1 cytokines, such as IL-2 and IFN-γ [36]. Although trans-resveratrol was most abundant in the GGP diet, *IFNG* tended to increase in the liver of PE-fed broilers. This set of evidence supports the hypothesis that it is the polyphenols’ chemical structure and not its dietary level that regulates the rate and extent of intestinal absorption, the nature of the metabolites circulating in the plasma, and hence, the organism response [37].

Interestingly, *IL8* was upregulated in the PE-fed broilers in all the investigated organs. IL-8 is also a pivotal chemokine of the host immune system against bacterial and viral infections and is considered to be a crucial pro-inflammatory mediator with chemotaxis properties [38]. Even though IL-8 synthesis appears to be crucial in the case of host infection, its upregulation in the absence of challenge may conceal a pro-inflammatory response.

The mRNA levels of *MAPK* in the bursa of Fabricius in the PE-fed broilers showed an upward trend. MAPKs involve at least six principal sub-family members, of which Erk and p38 *MAPK* are the dominants [39]. These sub-families regulate a broad spectrum of cellular functions, such as cell proliferation, differentiation, and survival in response to a wide variety of signals, including, but not limited to, growth factors and oxidative stress [39]. Remarkably, it is plausible to assume that cell type and stimulus have a powerful impact on the role of *MAPK* function [39]. Since scarce information exists aiming to ascribe *MAPK* upregulation on specific cellular functions, we have narrowed the focus of this research to highlight another insight for pro-inflammatory response in the PE-fed broilers.

Remarkably, the administration of individual polyphenolic compounds such as resveratrol [40], catechins [41], procyanidins [42], and quercetin [43] that are present in grape byproducts as well, have shown anti-inflammatory properties in animal and human experimental models. However, plant derivatives rich in polyphenolic compounds such as grape byproducts include more than one substance which may act synergistically or antagonistically when in vivo trials are concerned. More specifically, the insights for a low-grade pro-inflammatory response in the PE-broilers as was reflected in transcriptional level may be attributed to an adverse impact of polyphenol overload due to the synergistic combination of the 17 prevailing polyphenolic compounds in the stem extract [44]. On the other hand, plant-derived polyphenolic compounds are naturally found esterified in natural extracts, and their esterification severely impairs their absorption since intestinal mucosa, liver, and plasma do not possess esterases capable of hydrolyzing them [37].

Another mechanism that could be involved in pro-inflammatory signals in the PE-fed broilers may lie on their numerically higher final body weight. Hypothesizing that the heavier birds had also a higher percentage of white adipose tissue (WAT), pro-inflammatory cytokine upregulation may be attributed to the trigger effect of adipocytes on the immune cells [45]. Vice versa, the observed pro-inflammatory response was negligible, impairing broilers’ performance as has been previously observed when lipopolysaccharide-stressed broilers decreased their feed intake and daily weight gain [46].

The implementation of a high-throughput RT-PCR platform undoubtedly provided us a broad transcriptional perspective of the regulation of specific genes involved in the immune regulation. However, further research should be conducted on protein levels focusing on validating these preliminary outcomes. Several factors, such as various and complicated post-transcriptional mechanisms in translation from mRNA to protein, different half-lives of protein, and both mRNA and protein experimental limitations could result in discrepancies between mRNA and protein expression levels [47].

The immune regulation on a transcriptional level was not considerably impaired in the GGP- and WYC-fed broilers. These results are in accordance with the observations of Hasted et al. [32] where the majority of trials supplementing polyphenols in broilers diet had no effect on immune status. However, a suppression of the mRNA levels of the pro-inflammatory mediators would be expectable and desirable due to the high proportion of epicatechin [48,49] and b-glucans [50] in the GGP and WYC groups, respectively.

## 5. Conclusions

The exploitation of grape by-products as feed additives appears to be a promising strategy to improve waste valorization and to promote the circular economy. Although our preliminary study indicated that grape stems and wine lee extracts substantially improve breast oxidative status, this study unveils insights for a low-grade pro-inflammatory response when stem extracts were supplemented in broilers’ diet under a transcriptional perspective. Hence, wine lees are considered to be a valuable winemaking by-product capable of improving broilers’ antioxidative mechanisms and muscle oxidative stability without adversely affect birds’ performance and immune regulation. However, further studies are required to investigate the immunomodulatory properties of polyphenolic-rich grape by-products under challenging field conditions.

## Figures and Tables

**Table 1:** Sequences and relative positions of primers for target genes used in real-time qPCR.

Gene	Sequence	Amplicon bp	Accession No.*	References
*GAPDH*	F: 5′- GCTGGCATTGCACTGAATGAC -3′	113	NM_204305.1	[23]
	R: 5′- CACTCCTTGGATGCCATGT -3′			
*ACTB*	F: 5′- AGCGAACGCCCCCAAAGTTCT -3′	139	NM_205518.1	[24]
	F: 5′- AGCTGGGCTGTTGCCTTCACA -3′			
*TLR4*	R: 5′- ACCCGAACTGCAGTTTCTGGAT -3′	120	NM_001030693.1	[23]
	R: 5′- AGGTGCTGGAGTGAATTGGC -3′			
*MAPK*	F: 5′- GAACGTGCGCTTCATCTACG -3′	137	XM_040649449.1	
	R: 5′- CCACGGGCTTAAACGCTTTC -3′			
*NFKB*	F: 5′- GAAGGAATCGTACCGGGAACA -3′	131	NM_205134 131	[25]
	R: 5′- CTCAGAGGGCCTTGTGACAGTAA -3′			
*TNF*	F: 5′- CCCCTACCCTGTCCCACAA -3′	67	NM204267	[26]
	R: 5′- TGAGTACTGCGGAGGGTTCAT -3′			
*INFA*	F: 5′- ACTTCAGCTGCCTCCACACCTT -3′	92	AM049251.1	[23]
	R: 5′- CAGGAACCAGGCACGAGCTT -3′			
*INFG*	F: 5′- AACAACCTTCCTGATGGCGTGA -3′	89	NM_205149.1	[23]
	R: 5′- GCTTTGCGCTGGATTCTCAAGT -3′			
*IL1B*	F: 5′- TGCTTCGTGCTGGAGTCACCC -3′	98	XM_015297469.1	[23]
	R: 5′- GGCCGGTACAGCGCAATGTT -3′			
*IL2*	F: 5′- CGTAAGTGGATGGTTTTCCTCT -3′	161	NM204153	[27]
	R: 5′- GGCTAAAGCTCACCTGGGTC -3′			
*IL6*	F: 5′- AGCGAAAAGCAGAACGTCGAGTC -3′	107	XM_015281283.2	[23]
	R: 5′- GCCGAGTCTGGGATGACCACTTC -3′			
*IL8*	F: 5′- CTGGCCCTCCTCCTGGTT-3′	105	HM179639	[28]
	R: 5′- GCAGCTCATTCCCCATCTTTAC -3′			
*IL18*	F: 5′- GTTGTTCGATTTAGGGAAGGAG -3′	146	NM204608.1	[29]
	R: 5′- TCAAAGGCCAAGAACATTCC -3′			

* Ref Seq: NCBI Reference Sequence database.

**Table 2:** The total antioxidant capacity of grape pomace (GGP), dried wine lee extract (WYC), and grape stem extract (PE).

	Dietary Treatment		
	GGP	WYC	PE
FRAP	0.95 ± 0.07	2.6 ± 0.1	2.1 ± 0.2
DPPH	1.56 ± 0.01	1.846 ± 0.001	1.860 ± 0.007

Concentrations are expressed as mmol E-Fe(II): iron (II) equivalents/g extract for the FRAP assay and IC_50_ (mg Trolox equivalents/g extract) for the DPPH assay. All values are means ± standard deviation of three measurements.

## Data Availability

All data are available within the article.

## References

[1] FAO (2010). STAT-FAO Statistical Database. http://faostat3.fao.org.

[2] Brenes A., Viveros A., Chamorro S., Arija I. (2016). Use of polyphenol-rich grape by-products in monogastric nutrition. A review. Anim. Feed Sci. Technol..

[3] Filippi K., Georgaka N., Alexandri M., Papapostolou H., Koutinas A. (2021). Valorisation of grape stalks and pomace for the production of bio-based succinic acid by Actinobacillus succinogenes. Ind. Crop. Prod..

[4] Ferri M., Vannini M., Ehrnell M., Eliasson L., Xanthakis E., Monari S., Sisti L., Marchese P., Celli A., Tassoni A. (2020). From winery waste to bioactive compounds and new polymeric biocomposites: A contribution to the circular economy concept. J. Adv. Res..

[5] Brenes A., Viveros A., Goñi I., Centeno C., Sáyago-Ayerdy S.G., Arija I., Saura-Calixto F. (2008). Effect of grape pomace concentrate and vitamin E on digestibility of polyphenols and antioxidant activity in chickens. Poult. Sci..

[6] Righi F., Pitino R., Manuelian C.L., Simoni M., Quarantelli A., De Marchi M., Tsiplakou E. (2021). Plant Feed Additives as Natural Alternatives to the Use of Synthetic Antioxidant Vitamins on Poultry Performances, Health, and Oxidative Status: A Review of the Literature in the Last 20 Years. Antioxidants.

[7] Mavrommatis A., Giamouri E., Myrtsi E.D., Evergetis E., Filippi K., Papapostolou H., Koulocheri S.D., Zoidis E., Pappas A.C., Koutinas A. (2021). Antioxidant Status of Broiler Chickens Fed Diets Supplemented with Vinification Byproducts: A Valorization Approach. Antioxidants.

[8] Maraldi T. (2013). Natural compounds as modulators of NADPH oxidases. Oxidative Med. Cell. Longev..

[9] Park H.S., Jung H.Y., Park E.Y., Kim J., Lee W.J., Bae Y.S. (2004). Cutting edge: Direct interaction of TLR4 with NAD(P)H oxidase 4 isozyme is essential for lipopolysaccharide-induced production of reactive oxygen species and activation of NF-kappa B. J. Immunol..

[10] Liu W.C., Zhu Y.R., Zhao Z.H., Jiang P., Yin F.Q. (2021). Effects of Dietary Supplementation of Algae-Derived Polysaccharides on Morphology, Tight Junctions, Antioxidant Capacity and Immune Response of Duodenum in Broilers under Heat Stress. Animals.

[11] Liu W.C., Ou B.H., Liang Z.L., Zhang R., Zhao Z.H. (2021). Algae-derived polysaccharides supplementation ameliorates heat stress-induced impairment of bursa of Fabricius via modulating NF-κB signaling pathway in broilers. Poult. Sci..

[12] Surai P.F., Kochish I.I., Kidd M.T. (2021). Redox Homeostasis in Poultry: Regulatory Roles of NF-κB. Antioxidants.

[13] Burdick Sanchez N.C., Broadway P.R., Carroll J.A. (2021). Influence of Yeast Products on Modulating Metabo-lism and Immunity in Cattle and Swine. Animals.

[14] Shakoor H., Feehan J., Apostolopoulos V., Platat C., Al Dhaheri A.S., Ali H.I., Ismail L.C., Bosevski M., Stojanovska L. (2021). Immunomodulatory Effects of Dietary Polyphenols. Nutrients.

[15] Cuevas A., Saavedra N., Salazar L.A., Abdalla D.S.P. (2013). Modulation of Immune Function by Polyphenols: Possible Contribution of Epigenetic Factors. Nutrients.

[16] Ding S., Jiang H., Fang J. (2018). Regulation of Immune Function by Polyphenols. J. Immunol. Res..

[17] Myrtsi E.D., Koulocheri S.D., Iliopoulos V., Haroutounian S.A. (2021). High-Throughput Quantifica-tion of 32 Bioactive Antioxidant Phenolic Compounds in Grapes, Wines and Vinification Byproducts by LC-MS/MS. Antioxidants.

[18] Nitschke J., Modick H., Busch E., von Rekowski R.W., Altenbach H.J., Mölleken H. (2011). A new colorimetric method to quantify β-1,3-1,6-glucans in comparison with total β-1,3-glucans in edible mushrooms. Food Chem..

[19] Tsiplakou E., Mavrommatis A., Skliros D., Sotirakoglou K., Flemetakis E., Zervas G. (2018). The effects of dietary supplementation with rumen-protected amino acids on the expression of several genes involved in the immune system of dairy sheep. J. Anim. Physiol. Anim. Nutr..

[20] Qin N., Shan X., Sun X., Liswaniso S., Chimbaka I.M., Xu R. (2020). Evaluation and Validation of the Six Housekeeping Genes for Normalizing Mrna Expression in the Ovarian Follicles and Several Tissues in Chicken. Braz. J. Poult. Sci..

[21] Mavrommatis A., Mitsiopoulou C., Christodoulou C., Karabinas D., Nenov V., Zervas G., Tsiplakou E. (2020). Dietary Supplementation of a Live Yeast Product on Dairy Sheep Milk Performance, Oxidative and Immune Status in Peripartum Period. J. Fungi.

[22] Ramakers C., Ruijter J.M., Deprez R.H., Moorman A.F. (2003). Assumption-free analysis of quantitative real-time polymerase chain reaction (PCR) data. Neurosci. Lett..

[23] Csernus B., Biró S., Babinszky L., Komlósi I., Jávor A., Stündl L., Remenyik J., Bai P., Oláh J., Pesti-Asbóth G. (2020). Effect of Carotenoids, Oligosaccharides and Antho-cyanins on Growth Performance, Immunological Parameters and Intestinal Morphology in Broiler Chickens Challenged with Escherichia coli Lipopolysaccharide. Animals.

[24] Ahmadipour B., Hassanpour H., Khajali F. (2018). Evaluation of hepatic lipogenesis and antioxidant status of broiler chickens fed mountain celery. BMC Vet. Res..

[25] Hou X., Liu F. (2014). Polyphenol extracts from Punica granatum and Terminalia chebula are an-ti-inflammatory and increase the survival rate of chickens challenged with Escherichia coli. Biol. Pharm. Bull..

[26] Han H., Zhang J., Chen Y., Shen M., Yan E., Wei C., Yu C., Zhang L., Wang T. (2020). Dietary taurine supplementation attenuates lipopolysaccharide-induced inflammatory responses and oxidative stress of broiler chickens at an early age. J. Anim. Sci..

[27] Shang Y., Regassa A., Kim J.H., Kim W.K. (2015). The effect of dietary fructooligosaccharide supple-mentation on growth performance, intestinal morphology, and immune responses in broiler chickens challenged with Salmonella Enteritidis lipopolysaccharides. Poult. Sci..

[28] Deng J., Guo H., Cui H., Fang J., Zuo Z., Deng J., Wang X., Zhao L. (2016). Oxidative stress and inflammatory responses involved in dietary nickel chloride (NiCl2)-induced pulmonary toxicity in broiler chickens. Toxicol. Res..

[29] Paraskeuas V.V., Mountzouris K.C. (2019). Modulation of broiler gut microbiota and gene expression of Toll-like receptors and tight junction proteins by diet type and inclusion of phytogenics. Poult. Sci..

[30] Gungor E., Altop A., Erener G. (2021). Effect of Raw and Fermented Grape Pomace on the Growth Performance, Antioxidant Status, Intestinal Morphology, and Selected Bacterial Species in Broiler Chicks. Animals.

[31] Ebrahimzadeh S.K., Navidshad B., Farhoomand P., Aghjehgheshlagh F.M. (2018). Effects of grape pomace and vitamin E on performance, antioxidant status, immune response, gut morphology and histopathological responses in broiler chickens. S. Afr. J. Anim. Sci..

[32] Hasted T.L., Sharif S., Boerlin P., Diarra M.S. (2021). Immunostimulatory Potential of Fruits and Their Extracts in Poultry. Front. Immunol..

[33] Rahimifard M., Maqbool F., Moeini-Nodeh S., Niaz K., Abdollahi M., Braidy N., Nabavi S.M., Nabavi S.F. (2017). Targeting the TLR4 signaling pathway by polyphenols: A novel therapeutic strategy for neuroinflammation. Ageing Res. Rev..

[34] Yi R., Chen X., Li W., Mu J., Tan F., Zhao X. (2020). Preventive effect of insect tea primary leaf (Malus sieboldii (Regal) Rehd.) extract on D-galactose-induced oxidative damage in mice. Food Sci. Nutr..

[35] Chedea V.S., Palade L.M., Pelmus R.S., Dragomir C., Taranu I. (2019). Red Grape Pomace Rich in Polyphenols Diet Increases the Antioxidant Status in Key Organs—Kidneys, Liver, and Spleen of Piglets. Animals.

[36] Yusuf N., Nasti T.H., Meleth S., Elmets C.A. (2009). Resveratrol enhances cell-mediated immune response to DMBA through TLR4 and prevents DMBA induced cutaneous carcinogenesis. Mol. Carcinog..

[37] Pandey K.B., Rizvi S.I. (2009). Plant polyphenols as dietary antioxidants in human health and disease. Oxidative Med. Cell. Longev..

[38] Park S.H., Biswas D., Lingbeck J., Koo O.K., Ricke S.C. (2013). Enhancement of chicken macrophage cytokine response to Salmonella Typhimurium when combined with bacteriophage P22. FEMS Microbiol. Lett..

[39] Wang Y., Zeigler M.M., Lam G.K. (2007). The role of the NADPH oxidase complex, p38 MAPK, and Akt in regulating human monocyte/macrophage survival. Am. J. Respir. Cell Mol. Biol..

[40] Malaguarnera L. (2019). Influence of Resveratrol on the Immune Response. Nutrients.

[41] Fan F.Y., Sang L.X., Jiang M. (2017). Catechins and Their Therapeutic Benefits to Inflammatory Bowel Disease. Molecules.

[42] González-Quilen C., Rodríguez-Gallego E., Beltrán-Debón R., Pinent M., Ardévol A., Blay M.T., Terra X. (2020). Health-Promoting Properties of Proanthocyanidins for Intestinal Dysfunction. Nutrients.

[43] Li Y., Yao J., Han C., Yang J., Chaudhry M.T., Wang S., Liu H., Yin Y. (2016). Quercetin, Inflammation and Immunity. Nutrients.

[44] Mennen L.I., Walker R., Bennetau-Pelissero C., Scalbert A. (2005). Risks and safety of polyphenol consumption. Am. J. Clin. Nutr..

[45] Kern L., Mittenbühler M.J., Vesting A.J., Ostermann A.L., Wunderlich C.M., Wunderlich F.T. (2018). Obesity-Induced TNFα and IL-6 Signaling: The Missing Link between Obesity and Inflammation-Driven Liver and Colorectal Cancers. Cancers.

[46] Liu L., Qin D., Wang X., Feng Y., Yang X., Yao J. (2015). Effect of immune stress on growth performance and energy metabolism in broiler chickens. Food Agric. Immunol..

[47] Greenbaum D., Colangelo C., Williams K., Gerstein M. (2003). Comparing protein abundance and mRNA expression levels on a genomic scale. Genome Biol..

[48] Cheng A.W., Tan X., Sun J.Y., Gu C.M., Liu C., Guo X. (2019). Catechin attenuates TNF-α induced inflammatory response via AMPK-SIRT1 pathway in 3T3-L1 adipocytes. PLoS ONE.

[49] Afolabi O.K., Aderibigbe F.A., Folarin D.T., Arinola A., Wusu A.D. (2019). Oxidative stress and inflammation following sub-lethal oral exposure of cypermethrin in rats: Mitigating potential of epicatechin. Heliyon.

[50] Schwartz B., Vetvicka V. (2021). Review: β-glucans as Effective Antibiotic Alternatives in Poultry. Molecules.

